# Lived experiences of deaf parents: insights into pride, community, bilingualism, and barriers

**DOI:** 10.3389/fpsyg.2025.1537618

**Published:** 2025-06-17

**Authors:** Victoria St. Clair, Patrick Rosenburg, Evelyne Mercure

**Affiliations:** ^1^Centre for Brain and Cognitive Development, Birkbeck, University of London, London, United Kingdom; ^2^Institute of Cognitive Neuroscience, University College London, London, United Kingdom; ^3^Deafness, Cognition and Language Research Centre, University College London, London, United Kingdom; ^4^Department of Psychology, Goldsmiths, University of London, London, United Kingdom

**Keywords:** deaf parents, qualitative research, reflexive thematic analysis, bimodal bilingualism, sign language, communication, accessibility

## Abstract

**Introduction:**

Becoming a parent is a deeply personal process that changes the dynamics of one’s social and psychological worlds. Parenthood also exists within the context of cultural and, for those who give birth, medical backgrounds. Little is known about how those who identify as deaf experience navigating resources, medical and educational professionals, and support systems in parenthood.

**Methods:**

This study investigates the lived experiences of *N* = 37 deaf parents using reflexive thematic analysis.

**Results:**

Four primary themes were constructed from the data. The first was that many deaf parents felt a clear sense of pride and confidence in themselves and in their children. It was also obvious that deaf parents benefitted from the support of their family, friends, and their communities. A third theme was the importance and sometimes the challenges of navigating children’s bimodal bilingualism. Finally, deaf parents faced some common barriers: limited access to information and support, experiences of prejudice and discrimination, and the added stress and effort required to advocate for themselves and their children.

**Discussion:**

Overall, this study offers important insight into the experiences of deaf parents, shedding new light on the ways in which deaf parents access information and professionals related to child development, and on their experiences of parenthood overall.

## Introduction

Research on hearing parents’ experiences suggests that the transition into parenthood is a psychological, behavioral, and physical adjustment. Parents must navigate changes in their roles and responsibilities (Sanders et al., 2021). It is important that parents feel equipped with the knowledge required to comfortably negotiate the early days of parenthood (Deave et al., 2008). One’s experience of parenthood is likely related to their cultural contexts (Chen et al., 2019), biological experiences (Deater-Deckard et al., 2018), social identities (Fadjukoff et al., 2016), and on their specific role as a biological, non-biological, adoptive or foster parent (Pagé et al., 2017). In the case of deaf parents, experiences might be uniquely influenced by individual experiences of deafness, deaf culture and language use, in signed and/or spoken modalities. This study uses reflexive inductive thematic analysis to explore the experiences of deaf parents across the course of their children’s lives, from the early days of pregnancy, birth, and caring for a newborn through the later years of supporting children’s social and educational development.

Deafness is often discussed in terms of the “medical model,” which relates to the biological state of an absence of hearing, and the “social-cultural model,” which relates to the rich shared culture, history, and language of deaf people (Gregory and Hartley, 1991). In a medical sense, deafness occurs because of mechanical properties of the inner, middle, or outer ear, of the cochlear nerve, or both (Zahnert, 2011). A person’s audiological experience of being deaf varies widely depending on factors such as their degree(s) of deafness, when they were identified as deaf, and whether they use assistive devices like hearing aids or cochlear implants. Deaf people’s experiences are dynamic and related to the unique intersections of their many identities (e.g., ethnicity, race, sexual orientation, gender, etc).

Some deaf people use only spoken language(s), others use only sign language(s), and many use both (Kusters and Lucas, 2021; Kyle and Woll, 1988). Sign languages use the hands, face, and body to convey linguistic meaning and are equivalent in complexity to spoken languages (Bellugi and Fisher, 1972; Stokoe, 2005; Woll and Sutton-Spence, 2011). Sign language users are a linguistic minority with a long history of advocating for legal recognition of sign languages across the world (De Meulder, 2015; De Meulder et al., 2018, 2019; Hill, 2013). Deaf communities use hundreds of different signed languages around the globe (Fenlon and Wilkinson, 2015; Mathur and Napoli, 2010; Monaghan, 2003; Murray, 2008). Children of deaf signers may learn the sign language(s) of their parents. Throughout this study, deaf people’s language choices are discussed as specifically as possible to respect the many ways that a person’s audiological experiences and cultural identities interact with their language preferences, and the ways language use interacts with experiences of parenthood. Importantly, deaf people have both deaf and hearing children, though it is thought to be more common for them to have hearing than deaf children. Hearing children of deaf parents are often referred to as “children of deaf adults,” or “CODAs” (van den Bogaerde and Baker, 2016). CODAs are often raised multilingually and multiculturally, as they learn spoken language(s) from their hearing family and friends and, if their deaf parent uses it, sign language(s). Children who learn both a spoken and signed language are often called “bimodal bilingual” as they are learning two languages in two different modalities. Further, if a CODA’s parent is culturally Deaf, they may also be raised in the Deaf community, learning about Deaf traditions, values, history, and humor, and attending CODA camps and clubs and/or social events with their parents. CODA spoken and signed language development is also highly variable (Hofmann and Chilla, 2014; Pichler and Koulidobrova, 2015), which is unsurprising given the wide heterogeneity of the bimodal bilingual experience.

The social-cultural model of deafness—or “Deafhood” as “deafness” is a term associated with the medical model (Allen, 2015; Ladd, 2003, 2005)—posits that there is more to being deaf than the audiological experience. This view argues that deficit-centric views of Deafhood originate from the hearing-majority world and serve to marginalize deaf people (Bienvenu, 2016; Lane, 2005). The term “Deaf culture” refers to the diverse community of people who are proud of their Deaf identity (Ladd, 2003; Leigh et al., 2022; Maxwell-McCaw et al., 2019) and who feel tied to the rich history, traditions, and values of the Deaf world (Lane et al., 1996; Padden and Humphries, 1988, 2005; Schaffer and Kilanowski-Press, 2011). Extensive literature exists on the intricacies of Deaf culture (Holcomb, 2023), heterogeneity of Deaf experiences (Dunn and Anderson, 2020), benefits of being Deaf (called “Deaf Gain”; Bauman and Murray, 2009, 2013, 2014), disability justice pioneered by Deaf scholars (Robinson, 2017; Robinson and Henner, 2017), and transnational Deaf studies and Deaf anthropology (Friedner and Kusters, 2020; Murray, 2008).

Historically, a dichotomy has existed between those who identify as “capital ‘D’ Deaf,” meaning they identify as culturally Deaf, and “little ‘d’ deaf,” which tends to refer to those with the audiological identification broadly, regardless of cultural affiliation. In this paper, in reference specifically to culturally Deaf people or in discussion of previous research whose participants identify exclusively as “capital ‘D’ Deaf,” the word will be capitalized. Here, the term “deaf” is used as an inclusive umbrella term to include any person who is deaf, regardless of their level of deafness, cultural identities, or language choices. Debate exists about these classifications (Woodward and Horejes, 2016), but the use of “deaf” as a non-dichotomous category aligns with its use by organizations in the UK, where two of the three authors are based, such as the British Association of Teachers of Deaf Children and Young People and the National Deaf Children’s Society.

From the perspective of parents themselves, parenthood is shaped by one’s sociocultural contexts and access to resources. Mallory et al. (1992) examined the experiences of Canadian deaf parents and found that, overall, deaf parents were confident in their parenting. Of particular importance to this sample of deaf parents was their children’s communicative development and the ease of parent–child communication which was noted as a source of pride (Mallory et al., 1992). A national survey of American parents found that many Deaf parents report being happy, finding strength in their communities, and including Deaf traditions in their families (Olkin et al., 2006). Another qualitative study of Swedish children with deafblind parents found that children reported experiences similar to those with hearing and sighted parents (Huss et al., 2022). Children said there was “nothing weird or different” to them about having a deafblind parent and that they lived similar lives overall to their friends with hearing, sighted parents (Huss et al., 2022). Another study about the experiences of hearing children of deaf parents in Ireland found similar themes: “it was really normal” (Heffernan and Nixon, 2023).

A common challenge reported by deaf parents are situations that undermine their role as parents. Several studies describe parents’ concerns about parent–child role reversals (Heffernan and Nixon, 2023; Huss et al., 2022; Mallory et al., 1992). Hearing children of deaf parents are sometimes asked to act as interpreters for their parents in difficult contexts, which Mallory et al. (1992) found sometimes made deaf parents feel uncomfortable. A more recent thematic analysis of the experiences of deaf parents in the Czechia reported that children, sometimes as young as 5 or 6 years old, often interpreted for their parents in situations they were not fully prepared to understand (e.g., for doctor’s appointments; Klimentová and Dočekal, 2020). Research has also suggested that deaf parents are sometimes excluded by their children’s schools (Mallory et al., 1992), with one study reporting that 22% of Deaf parents experienced difficulties communicating with their children’s school staff (Olkin et al., 2006). One study of Portuguese Deaf parents also revealed frequent encounters with communication breakdowns and difficult attitudes from children’s schools (Barbosa et al., 2023).

It has also been shown that deaf people tend to face more barriers than hearing people do in medical settings. For example, James et al. (2021) investigated patterns in Deaf American signers’ experiences of emergency medical care. The results suggested that communication can be stressful, frustrating and time-consuming (James et al., 2021), and Deaf patients reported a general lack of cultural sensitivity from providers. Similar patterns have been reported among deaf childbearing women in medical settings. Two quantitative, comparative studies of deaf and hearing childbearing American women found that hearing women reported significantly higher levels of satisfaction with prenatal care, communication, and feelings that their physician cared for their health compared to deaf women (O’Hearn, 2006; Steinberg et al., 2004). Another qualitative study found that pregnant deaf women in America experienced frequent communication barriers in medical contexts (Ratakonda et al., 2024). When suitable accommodations were not made to improve accessibility, deaf mothers reported feeling confused and disempowered (Ratakonda et al., 2024).

It is also likely that deaf parents may not be provided with resources they feel are both accessible and relevant to them. For example, for a profoundly deaf, signing parent, advice about how to support children’s spoken language development may not feel useful to support their parenting goals. Formats of materials may make information inaccessible. Written-source materials like books, articles, magazines, forums, and leaflets, especially those that contain jargon, are not always accessible to deaf people. Chandanabhumma et al. (2024) investigated differences in the way deaf and hearing people accessed online health information. They found that the ways Deaf participants accessed online health information was different to hearing counterparts and that they had varying degrees of success. For example, Deaf participants said they had difficulty identifying specific information they wanted, negotiating medical jargon, and finding information that was displayed visually (Chandanabhumma et al., 2024; see also Mallory et al., 2019).

Overall, the limited research available suggests that deaf parents are likely to have specific experiences that are intertwined with their audiological, social, cultural, and linguistic circumstances. However, the extent to which these factors influence deaf parents’ early days of accessing prenatal care to, in later years, supporting their children’s school integration and social development remain largely unexplored. Research has yet to investigate heterogenous samples of deaf parents, including those who live in vastly different environments, who may or may not have given birth themselves, or who indicate a wide variety of language preferences (e.g., native signers, spoken-language users, etc). Moreover, no research to date has systematically investigated the advice deaf parents receive about their language choices and their children’s language development. This study aims to fill these gaps to both extend existing conclusions to new contexts and to uncover novel themes. Three specific domains – deaf parents’ confidence, access to information, and commonly encountered language attitudes – served as the starting place for survey construction, though specific research questions shifted after initial engagement with the data (see “Analytical Approach” below). Overall, this research promotes an inclusive perspective about parents’ experiences to reveal deaf-derived strategies that could benefit all parents, reduce inequalities between deaf and hearing parents, and diversify current academic and practical knowledge more broadly.

## Methods

### Researcher positions and reflexivity

The research team brings to their analysis many different identities and experiences that inform their ontological and epistemological assumptions (Braun and Clarke, 2022). These theoretical orientations are important to acknowledge (Elliott et al., 1999) as they inform each individuals’ roles in the project, their motivation for involvement, and their approach to the research process. VS is a white, hearing, American female who emigrated to the UK 7 years ago. Her first language is English, her second is American Sign Language (ASL), and her third language is British Sign Language (BSL). She is a child development researcher with a degree in ASL and Deaf Studies, a master’s and doctorate in cognitive neuroscience, and an academic background in stigma psychology. Her work relies primarily on quantitative methods. As someone who is not deaf nor a parent herself, VS is firmly situated as an outsider in the community studied here (Elliott et al., 1999). However, she has lived, studied, and worked in culturally Deaf communities for over 10 years, and she works as a trainee BSL interpreter. Her involvement in culturally Deaf spaces with sign language users will have influenced the writing of this manuscript, construction of the research methods, and interpretation of the results.

PR is a white, Deaf, American male, a qualified teacher, and a researcher with a doctorate in education. He has experience teaching research methods and has supervised several theses and research projects in both the US and UK. He emigrated to the the UK and worked as a teacher of the deaf. He currently works as a post-doctoral researcher looking at the role of visual input in reading development in young deaf children. His professional journey has been shaped by his lived experience as a Deaf individual as well as his involvement in the deaf community as an advocate and educator. He has delivered training sessions and workshops for professionals working with the deaf people, addressing topics such as language development, communication issues, and other deaf-related issues. His contribution to this study is guided by his own experiences and those of other deaf individuals. This perspective ensures that the research is grounded in the realities and narratives of the population it seeks to represent.

EM is a white, hearing, Canadian and British female. She is a quantitative researcher and lecturer in psychology. She lived in Europe for 20 years and is now based in the USA. She has studied speech language therapy, neuroscience and psychology. EM is bilingual in French and English, and she holds a Level 2 certificate in BSL. EM has been studying neurocognitive development in hearing children of deaf parents for the last 12 years. While conducting her research, she informally discussed lived experiences with some of her research participants, and this triggered her interest in investigating this topic further. EM is a parent, but as a hearing person, she considers herself to be mostly an outsider to the studied population. Her contribution to this study will be influenced by her personal experiences of navigating healthcare and educational systems as a parent in the United Kingdom, France and the USA and by her prior research on neurocognitive development in hearing children of deaf parents.

### Data collection

Participants were originally intended to be recruited from the USA and UK. However, it quickly became apparent on social media that deaf parents from other countries who used written English wanted to participate. The criteria were changed 5 days after recruiting began to be open to any deaf parent who felt comfortable reading English, which resulted in one participant from Australia. This meant there were no restrictions on which signed languages participants used (if any). The wide geographic spread of participants was well-suited to answer the research question, as the team were interested in understanding the lived experiences of deaf parents with a broad view. The goal was to construct themes common to deaf parents in a diverse sample, regardless of their country of residence, their language preferences, their children’s hearing statuses, or the sign language(s) they used. Other inclusion criteria were that parents were over the age of 18 years old and that they had at least one child under the age of 18 years old. There were no exclusions based on parents’ gender, aspects of the parent’s deafness (e.g., level, age of identification, cultural identity, language preferences), their biological relationship to their children, their children’s hearing statuses, or the number of children in the family.

The survey was open to participants from July to October 2023. Information about the study was distributed via personal networks and on social media platforms (i.e., X, Instagram, and Facebook), especially in groups intended to support deaf parents. It was also distributed on deaf-led media outlets (e.g., Limping Chicken), charities (e.g., SignHealth in the United Kingdom, Hands & Voices in the United States), and forums (e.g., Global Coalition of Parents of Children who are Deaf and Hard of Hearing). It was distributed via email listservs of regional deaf societies, social clubs, and schools spreading from California, USA to Berkshire, England. Efforts were also invested in general parenting network websites and groups that were not deaf-specific but of which deaf parents may have been part. Recruitment efforts included written English (e.g., formatted social media posts and captions) but were always presented alongside BSL or ASL recruitment video produced by PR.

The survey contained seven Likert scales, three yes/no questions, and 11 open text field questions that asked about deaf parents’ experiences in a wide variety of domains, such as their confidence in various topics, how they engaged with prenatal information and parenting support groups, and their experiences attending medical appointments. Questions also asked whether parents received advice about their children’s language development and the nature of their engagement with their children’s schools (see Appendix for list of questions). To ensure maximal accessibility, participants were given the option to participate either by recording responses in written English, by attending a semi-structured interview where interpreters would be provided, or both. This structure allowed participants to take part in their preferred language, either English or sign language. Most participants chose to reply to the survey in written English. Prompts were written with a Flesch Reading Ease score of 70/100 to be easily understood by average 13- to 15-year-olds (Flesch, 1948), which is similar to the average reading age reported in American Deaf adults (e.g., McKee et al., 2016). Each section of the survey had instructions in English, BSL, and ASL.

### Analytical approach

A reflexive, inductive thematic analysis approach was used (Braun and Clarke, 2022). In a reflexive approach, the researchers’ subjectivity is viewed as a resource that can enrich the research rather than something that needs to be strictly controlled (Gough and Madill, 2012). Throughout this work, the researchers attempt to acknowledge their individuality as an important part of the knowledge generated and situate the present findings appropriately. The coding process took place over 7 months, allowing the researchers to both engage and distance from the data to allow insights to develop (Braun and Clarke, 2022). As a first step, VS and EM generated codes for a subset of the data and met to discuss conceptual approaches to coding. They then independently coded the full dataset and generated initial themes. As the team met to refine and synthesize final themes, research questions grew increasingly focused (Braun and Clarke, 2022). It became obvious that imposing a three-fold structure focusing on accessibility, language attitudes, and parental confidence was insufficient; many parents discussed their experiences as dynamic and interrelated across these domains. The question then shifted toward understanding what parents described as “positive” compared to “negative” experiences, but this still overly simplified a rich and nuanced dataset. In final form, the research questions were: (1) How do deaf parents find parenting support and information, and what are the common barriers to access? (2) How do deaf parents view their children’s language development? (3) How do deaf parents feel about their roles as parents? Overall, this process allowed for refinement of research questions alongside iterative theme construction, grounded in the data and informed by the researchers’ previous experiences in the relevant communities (Braun and Clarke, 2022). VS checked the final themes against the full dataset to ensure they were indeed data-driven and answered the research questions. Debriefing and reflexive notetaking took place throughout the process. Quotes in the results section were selected by both VS and EM.

In the surveys, four Likert scales asked participants to rate their experiences on a categorical scale (e.g., “Very difficult” to “Very easy”). Descriptive statistics for these questions are included at the beginning of the primary results section to provide additional background to interpret the qualitative data. Ethical approval was granted for surveys and for semi-structured interviews by Goldsmiths, University of London’s Research Ethics and Integrity Sub-Committee (#1748).

### Participants

Responses were collected from a total of *N* = 39 participants using a Qualtrics survey. Of those responses, *n* = 25 chose to respond in English only, *n* = 3 expressed interest in participating in signed language, and *n* = 11 expressed interest in both. Those who indicated interest in a remote semi-structured interview were contacted with more information. Only *n* = 1 participant attended the semi-structured interview in BSL. Responses from this participant were transcribed using OtterAI and coded alongside survey responses. Data from a final sample of *N* = 37 participants are presented here. Thirteen participants lived in the United States (*n* = 1 from Arizona, *n* = 4 from Maryland, *n* = 1 from Massachusetts, *n* = 1 from Minnesota, *n* = 2 from New York, *n* = 1 from Texas, *n* = 1 from Virginia, and *n* = 2 state not specified). A further *n* = 22 lived in England and *n* = 1 from Wales. There was also *n* = 1 participant who lived in Australia. See Table 1 for participant information and language preference details.

**Table 1:** Participant demographics by country of residence.

	America (*n* = 13)	United Kingdom (*n* = 23)	Australia (*n* = 1)
Parent demographics			
Age			
18 to 24	*n* = 1	*n* = 1	-
25 to 34	*n* = 2	*n* = 7	-
35 to 44	*n* = 9	*n* = 10	-
45 to 54	*n* = 1	*n* = 4	*n* = 1
NA	-	*n* = 1	-
Gender			
Men	*n* = 5	*n* = 3	-
Women	*n* = 7	*n* = 20	*n* = 1
NA	*n* = 1	-	-
Family Composition			
Number of children			
One child	*n* = 7	*n* = 9	-
Two children	*n* = 4	*n* = 11	-
Three children	*n* = 1	*n* = 2	*n* = 1
Four children	*n* = 1	-	-
NA	-	*n* = 1	-
Hearing status of children			
All children hearing	*n* = 8	*n* = 16	*n* = 1
All children deaf	*n* = 1	*n* = 4	-
Some children hearing and some deaf	*n* = 4	*n* = 4	-
Age of children			
0 to 3	*n* = 3	*n* = 13	-
4 to 7	*n* = 7	*n* = 13	*n* = 1
8 to 11	*n* = 6	*n* = 4	-
12 to 18	*n* = 4	*n* = 4	*n* = 1
19 or older	*n* = 1	*n* = 3	*n* = 1
NA	*n* = 1	*n* = 1	-
Number of caregivers per child			
One	*n* = 1	*n* = 2	-
Two or more	*n* = 12	*n* = 21	*n* = 1
Daily language(s) used with children			
English only	*n* = 1	-	-
American Sign Language only	*n* = 4	-	-
English and American Sign Language	*n* = 8	-	-
British Sign Language only	-	*n* = 3	-
English and British Sign Language	-	*n* = 19	-
British and French Sign Language	-	*n* = 1	-
English and Auslan	-	*-*	*n = 1*

Age of children indicates number of children overall that families reported for each age range. Auslan is the signed language used by most signers in Australia. Where parents indicated they had a child over the age of 19, they also reported having other children younger than 18 and therefore met the study inclusion criteria.

**Figure 1:**
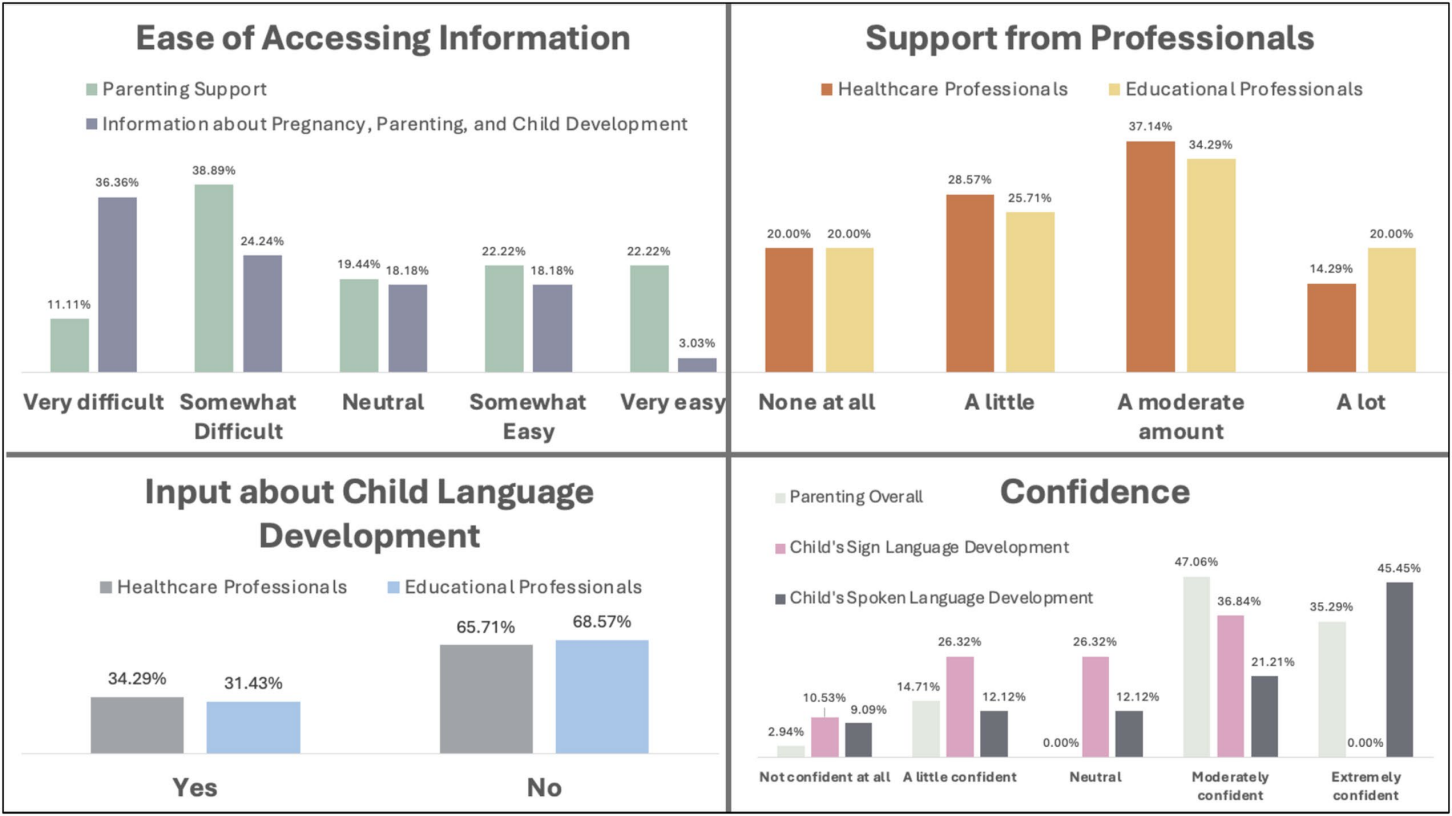
Descriptive statistics of parent-report scales. All proportions calculated as the number of responses for each option out of the total number of responses provided.

Quantitative data from four Likert scales and yes/no questions were collected from English survey respondents (*n* = 36). Descriptive statistics of this data are presented here to support interpretation of the qualitative themes (see Figure 1).

## Results

Parents’ responses to Likert scale questions can be found in Figure 1. Ratings indicated that finding accessible information about pregnancy, parenting, and child development was particularly difficult (60.6% found it very or somewhat difficult). Finding accessible parenting support groups was also challenging, though slightly less so (50% found it very or somewhat difficult). Most parents say they received a moderate or a lot of support generally from both healthcare (51.43%) and educational (54.29%) professionals. When it came to input about their children’s language development specifically, most did not receive input from healthcare (65.71%) or educational (68.57%) professionals. Finally, most parents were moderately or extremely confident in their child’s spoken language development (66.66%), but fewer were confident in their child’s sign language development (36.84% moderately confident, none extremely confident). When it came to parenting overall, most parents were moderately or extremely confident (82.35%) (Figure 1).

### Theme 1: pride and confidence

Many deaf parents described feeling proud of their children and confident both in their children’s growth and in their own skills as parents. Many specifically described their children as enjoying school, participating in class and extracurricular activities, making good friends, and thriving with supportive teachers. Several referenced children’s high levels of achievement in specific subjects, such as this parent:

The last two years, [my son] had been placed in Honors English class, which shows that the school district also has confidence that he is very, very capable. I am very proud of [him].

Many deaf parents reported feeling optimistic about their children’s future successes. Several said they worked hard to provide optimal learning environments for their children because they believed their children could succeed with the right support. One parent said, “[My son] is on par in his core subjects and option subjects…. However, if he works hard he could achieve higher!”

Most parents were also confident in themselves as parents (82.35% moderately or extremely confident, see Figure 1). Some described that their confidence grew with experience, while others mentioned feeling more confident with their second or third or fourth children than they did with their first. Several participants had careers working with children, and their professional experiences helped them to feel confident as parents. Overall, parents reported being proud of their children’s development, optimistic for their future achievements, and confident in themselves.

### Theme 2: it takes a village

Many deaf parents garnered support from family, friends, and their surrounding communities to guide their parenting decisions and their children’s development. Links with deaf parents or parents of deaf children were especially valued. One parent said:

My son goes to a nursery where there are a group of deaf parents or hearing parents with deaf children so we all group together. The saying of “[the] more people, the greater power they have” is true.

Another said:

I think as I come from a deaf family and have a strong network, I am more confident in knowing what I need to do for my children. The saying of “it takes a village” to bring up a child is very true in my case. We all bring different knowledge to the table and use our networks to make sure things happen.

Some who had deaf parents themselves described looking to their parents as role models. Deaf parents also experienced unhindered access to information from other deaf people. As put by one parent, “I just rely on information from deaf community. I did the research myself and talk with my deaf friends - direct information in our language.” Some deaf parents of deaf children referenced putting their child in a deaf school and described how, as a parent, they benefitted from fully accessible resources (e.g., parent-teacher meetings with deaf, signing teachers) to understand their child’s progress.

Many emphasized the importance of a community with same-aged children, particularly to normalize and validate difficult experiences. For example:

When you have a 9- to 10-month-old, you become very involved [in] conversations about sleep patterns and weaning and all things that are so important at that time. I remember talking with friends who have babies [and saying to them], “I did not sleep last night” and they’d say they did not either. That connection really helps you feel like you are not alone and [your struggles] are a normal part of raising children.

Some parents expressed difficulties in finding support and friendship within their community and therefore experienced loneliness (18 codes of the 43 total for Theme 2). Some deaf parents found it difficult to connect with parents in spaces created by hearing parents because of communication barriers, or because hearing parents did not appear they wanted to or knew how to include a deaf person. Online support was sometimes viewed as less powerful than having a community nearby, though remote groups were considered by some to be more accessible than face-to-face options. Particularly for first-time deaf parents, the lack of community had a negative impact. As one parent said, “It’s hard doing everything on my own.”

Some parents of school-aged children expressed that their children were excluded in nursery and school settings. Several parents said communication barriers between them and other hearing parents meant they were not included in the arrangement of children’s play dates, which resulted in their children being excluded from peer groups. Some parents felt excluded from activities in which they were keen to take part. For example:

I had to read lips a lot which was exhausting. [The school] had mandatory quarterly meetings… [but] they had no budget to pay an interpreter. I found one who would [work] pro bono two times but then nothing after that. [The school] said I did not have to attend the meetings anymore, but that [meant] I lost my opportunity to vote and contribute to the co-op’s program.

Another parent described their experience of exclusion within their family:

The only area [I’m] not feeling confident [in] is knowing what my kids are chattering/arguing about. They keep pointing to each other saying ‘she started it!’ Then I get frustrated I’m getting the runaround or [that] one kid lied. It diminishes my efforts to guide and discipline. My spouse is hearing and can hear what’s going [on] and intervene before anything becomes an issue. [The] kids have started favoring him.

### Theme 3: bimodal bilingualism

Many deaf parents in this sample felt they belonged to both “worlds” – hearing and deaf. For example:

[We are] fourth deaf generation on my side and sixth deaf generation from my wife’s side. We are living in the deaf world but our careers in the hearing world. We have positive attitudes in both ways.

Nearly all parents were also bilingual in spoken/written and signed languages, meaning they were bimodal bilinguals, using two languages in two different modalities. Most parents used a sign language with their children (see Table 1). The ways that parents navigated bimodal bilingualism was related to their child’s hearing status: some with deaf children referred to written English and signed language bilingualism while others with hearing children discussed spoken (and written) English and signed language bilingualism. Many deaf parents described using intentional bilingual strategies in the home, such as instituting “voice off” days to encourage children to practice signing, or swapping language days with their partner so children were exposed to both languages by both parents. As one parent said:

[My mom] and I worked out a regular schedule of twice a week video chat with my son from 6 months to 6 years old. This was to develop their grandmother-grandson relationship and also to expose [him to the] English language.

Deaf parents who had hearing parents or siblings themselves, or those with a hearing partner, said their children were exposed to spoken language from those family members. Hearing family members were often cited as reliable informants about children’s spoken language development. For example:

[I am] relying on my hearing partner to inform me the progress of [my children’s] spoken language development and a sister in law who is a speech language pathologist. [She] would check in and let me know if there was any concern. Some teachers were good with letting me know as well.

Parents felt their strategies worked to a variety of different degrees. Some said their children used sign language every day, were equally comfortable in spoken and signed language, and could switch between languages easily depending on their conversational partners. For some, sign language use was referenced as a point of pride for both parents and for children: “[My son] has proudly shared with others that his first language is ASL.” Others described struggling to promote a spoken-signed bilingual approach with their hearing children, especially in the case of deaf parents who were the only deaf person in their child’s family. For example, one participant said of her daughter, “Because she had limited access to sign language away from me, she [knows how] to use signs with me but not with other deaf people.”

Some participants worked hard to provide their children with what they felt was sufficient exposure to sign language but worried it might not be enough for their children to feel equally comfortable using both speech and sign. Parents also felt less confident in their children’s signed language development compared to their spoken language development overall. Likert scale ratings showed that 67% of parents were somewhat or very confident in their children’s spoken language development, while 37% were somewhat or very confident in their children’s sign language development (see Figure 1).

Some parents described how, in a hearing- and spoken-language-majority world, there are many settings for children to be exposed to speech and practice their spoken language skills (e.g., hearing-dominant nurseries, educational YouTube videos). Several parents also mentioned that when they were young, their children copied how they pronounced certain words. It was never referenced with concern, but instead with humor and/or curiosity. For example:

[My children] all speak well. When they were younger, they did have a deaf twang with some of their pronunciations, but when they started school, they quickly picked up the right pronunciations.

Deaf parents sometimes faced difficult attitudes about their role in their children’s early language development. One parent said, “I got the feeling that the daycare had made the judgement that I ‘was not doing my job as a parent’ just because I used ASL mostly with my son.” A number of deaf parents were directed to speak as much as possible to their children, despite not using spoken language themselves, and to analyze babies’ cries, though they could not hear them. Several parents said they only received information about children’s spoken language development, and that they were left feeling unsure about how their children’s use of sign language fit into this picture.

Several parents said they were met by a lack of recognition of the bilingual status of their child from their school and that schools “[did] not get what bilingual really meant.” The consequences included some parents not receiving appropriate, bilingual-specific support from schools and, quite often, facing ignorance about sign language and deafness that left some feeling they and their children were misunderstood. For example, one parent describes:

I placed my son in daycare at age 2. They reported concern [about] him not talking at age level even though he used ASL. It became clear that the daycare did not realize ASL is a language because they were focusing on the fact he was not using English. They had [an] Early Intervention agency get involved. The Early Intervention agency and I went back and forth. They eventually understood that the language used at home was not English and that they could send a support staff to the daycare to expose my son to the English language.

Many parents also described asking schools to co-develop strategies to support their children’s bilingualism, which, for some, was met with resistance or misunderstanding. Some reported that schools focused almost entirely on spoken language, did not understand the experiences of deaf parents or their children, and/or disregarded Deaf culture and sign language. Negotiating these attitudes was particularly difficult for some parents who felt less confident about their children’s bilingualism. For example:

Our children can understand our signing but will not sign back to us. Nursery do not see this as a problem despite us asking if they could use fingerspelling when doing their phonics. It’s clear they do not use any fingerspelling.

Overall, many deaf parents were invested in their children developing the language skills and cultural awareness required to integrate into both deaf and hearing environments. At home, some parents felt they could foster bimodal bilingual environments that best suited their goals with their children. However, some parents felt their children’s medical professionals and school staff gave inflexible advice rather than adapting to fit the context of their children’s bimodal bilingual language environments, which was described by some parents as frustrating. Others felt the wider community did not recognize the importance of their child’s cultural position or status as a bimodal bilingual, which led to some feeling they and their children were misunderstood.

### Theme 4: barriers

Many deaf parents described facing common barriers, including inaccessible information and services, experiences of prejudice and discrimination, and expenditure of extra energy and stress. When deaf parents had positive experiences that contrasted these typical barriers, they tended to describe themselves as “lucky.” They said they were lucky to be able to access information in written English, lucky their council had good resources, and lucky that hospitals provided interpreters for their medical appointments. It seemed clear that deaf parents found it more unusual not to encounter these barriers than they did to encounter them.

The most prominent barrier, for all deaf adults in all areas of parenthood, was inaccessible information and parenting-related support. Half of parents reported that parenting support resources were very or somewhat difficult to access, and 61% said information about pregnancy, parenting, and child development was very or somewhat difficult to access (Figure 1). As one parent put it, “If I needed support of professionals, this simply wasn’t accessible.” The most common reason was lack of sign language interpreters. There were anecdotes about hospitals and schools refusing to provide interpreters, saying interpreters would be provided when they were not, or making last-minute appointment changes that meant interpreters were not present. One parent said of her midwives,

They text me last minute or even turn up to my home early or at a different time [than was] agreed expecting me to write [to communicate]…. They lack the awareness of the difficulties [of] booking a BSL interpreter and [do] not seem [to think] that they need to adapt to me instead of me adapting to them.

Particularly, but not exclusively, in classes run by volunteers or private organizations, some deaf parents were told there was no budget for sign language interpreters and that they would need to pay for their own. Environments themselves were sometimes described as inaccessible, especially in-person parenting classes in noisy local community centers with poor acoustics. One parent said of baby groups, “they have been very difficult to participate in because it’s difficult to hear/lipread with distractions of babies crying and squealing in the background.” Several said that while group classes were too noisy without interpreters, they would have been able to follow in a more expensive one-on-one setting if they could have afforded it.

Some deaf parents handled the lack of interpreters at appointments, meetings, and classes by choosing not to take part despite wanting to, while others attended despite knowing they would not enjoy full access. Many parents reported missing out on support groups on specific topics they wanted to learn about, such as gestational diabetes, government programs for parents (e.g., UK Sure Start Maternity Grants), pre-natal vitamins, miscarriages, and in-vitro fertilization, because they would not have had access to the information without an interpreter. Attending appointments and events without interpreters was generally ineffective for deaf parents. One said they, “tried pregnancy yoga without interpreters and quit after two sessions. Tried breastfeeding group one time without interpreters.” In the words of one parent, attending without interpreters “makes me very anxious.” A similar feeling was expressed about lack of access to medical appointments with their GP (“General Practitioner” in the UK equivalent to PCP, “Primary Care Physician” in the US). For example: “I get anxious waiting to see if I can catch my name being called across the rooms.” Then, in appointments without interpreters, one deaf parent describes the need to “[adjust] to my second language to communicate with [my GP] via pen and paper.” Some mothers found it difficult to secure interpreters for their births. One mother said she endured a “difficult birth” without interpreters and “had no clue when my child was born.” In the experience of another parent,

I got overwhelmed in baby weighing session which took place in a hall off a public library with no BSL interpreter. Lots of parents talking, babies crying. I could not hear when it was our turn and could not understand what midwives were trying to say. I needed to watch my baby. I could not handle my baby and try to lip read at the same time. I left with no information…. [Then I made] mistakes [about] how much formula milk I gave to my baby, [resulting in] trips to A&E [“Accident & Emergency” equivalent to the ER, “Emergency Room” in the US]. [I had] no accessible information until [the] doctor wrote down weight ratio for milk.

Overall, many deaf parents relied on English materials for information about parenthood. These included books, magazines, blogs, parenting forums, news and government websites, and leaflets. However, these were described as difficult or impossible to access for those “whose first and only language” was sign language. Leaflets sometimes included hotlines to phone for more information or contained a lot of medical jargon. One parent stated, “nothing was available in BSL…. I would have questions about the information in terms of clarity and content specific to my children, but I was not be able to follow this up with professionals because no BSL provision was provided.”

Parents felt communication barriers were reduced when professionals asked parents about their communication preferences and adapted their strategies. As one deaf parent said, “I did not particularly need accessible (i.e., BSL) classes. [I] just [needed them to be] willing to work with me when I could not hear or [if I] misunderstood something, and that was generally what I got.” Many professionals, like local midwives and teachers, adapted well and were described by parents as “fantastic,” “brilliant,” and “amazing.” Deaf parents appreciated the support of deaf or hearing healthcare professionals who were either conversational in sign language or aware of deaf cultural norms. In these instances, deaf parents reported feeling respected, accepted, and safe.

However, many deaf parents described frequent encounters with those who held prejudicial attitudes about deafness. Some deaf parents were told by medical professionals that they should not be left alone with their children while others were treated as though they were uneducated about language and/or child development despite holding degrees in these topics. Lack of deaf awareness about how to communicate with deaf people was widespread, as this participant describes:

Many [school] staff members do not believe I’m deaf because I have coping strategies to hear. When I say I’m deaf and explain what would help (lip reading, not shouting, etc.) I’m ignored. Many people continue to shout and exaggerate their lip patterns.

Reports of discrimination were also frequent. Some deaf parents described being given fewer details by nursery or medical staff than hearing parents. One parent illustrated this in the following example:

Both myself and [my child’s] mother are deaf - I would see a lot of discrimination towards us in comparison to the hearing parents. There was one occasion where the school needed to get hold of us - they had the wrong phone number for me (their fault), and [their] mother was not contactable due to her work. Because of this, I had to meet the Designated Safeguarding Officer with the threat of social services in the background. However, other hearing parents who went through similar situations did not have to experience this.

Some parents experienced doctors expressing concerns about them having children in case their children were also born deaf. Others felt patronized, such as when one parent was told that birth would be painful. Some also described feeling overlooked:

There have been a few occasions whereby the medical professionals would not listen to us as the parents but choose to listen to the interpreter instead. This made us feel disregarded and belittled.

In a similar vein, one parent described that their child was put in a situation where they were expected to lead communication during medical appointments:

As the kids became older, they would communicate with the professionals themselves and relay information to [me]. The priority is to access healthcare, with communication second, so it was very frustrating and a breach of the Equality Act. I was always uneasy [about] my children leading communication at these appointments.

Some parents reported feeling “exhausted” and burnt out as they needed to advocate so often for themselves and their children. One deaf parent knew their deafness was genetic but needed to fight to get their children’s hearing checked, which resulted in a delay of their child’s identification as deaf. Several mothers report feeling acute stress in the months before the birth of their children because they needed to convince hospitals to provide interpreters for their deliveries. School environments were described by some as tiring because, as their children changed classrooms or schools, old challenges were renewed. Some parents anticipated “working hard for anything we may want or need” in each new academic year, and others advocated for their children for long periods of time before seeing changes. One parent said: “I brought up my concerns several times over 3 years…. it was only last 2 months (out of her 4.5 years nursery journey) things improved.” Many needed to re-contact and re-request services (e.g., interpreters to be provided at parent meetings) many times before seeing a result, if they saw one at all.

Another way in which parents expended extra energy was fielding requests to educate others about deafness. Some said that schools in particular were open-minded and curious about deafness but placed the burden on them (the deaf parent) to teach the staff sign language and educate them about deaf culture. One parent said, “I felt that I am educating them rather than another way around when it comes to hearing, language development, and child safety with deaf parent at home, etc. [It’s] tiring.” While open-mindedness and curiosity about deafness were viewed positively overall, the context was important to many deaf parents. Those who were seeking support from professionals and were then asked to educate those professionals in times of vulnerability or stress described feeling frustrated and tired.

## Discussion

**Figure 2:**
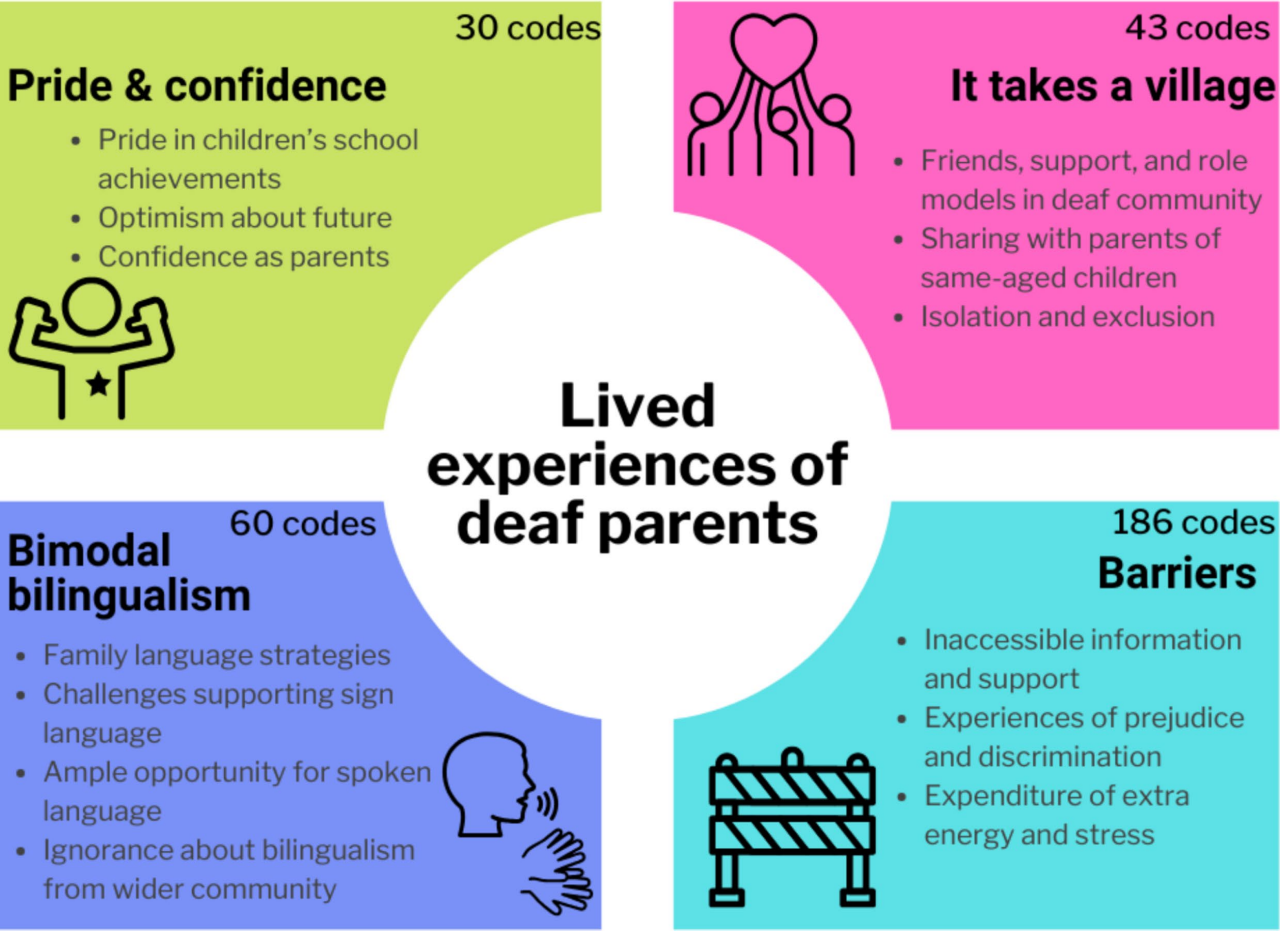
Summary of findings. Schematic of four primary themes.

This study provides new insight into the lived experiences of deaf parents. Overall, deaf parents expressed confidence in themselves and their children, gratitude for the role of community support in raising their children, and said they were invested in their children’s bimodal bilingualism. Many also said they repeatedly experienced barriers (Figure 2). The themes constructed build on previous research by investigating lived experiences of a diverse group of deaf parents and in a wide variety of social, educational, and medical settings across the course of children’s lives. Overall, this study serves to highlight the many strengths and positive experiences of deaf parents while also providing important direction for professionals and organizations to reduce barriers for deaf parents.

### Theme 1: pride and confidence

For parents of school-aged children, there was a sense of pride about children’s educational achievements. Some described fostering supportive home environments to help children learn, while others said they were both proud of their children’s past achievements and optimistic about their futures. Studies suggest that parents’ mindsets about the malleability of intelligence relates to children’s educational attainment (Haimovitz and Dweck, 2016; Matthes and Stoeger, 2018). One study by Song et al. (2022) found that parents who believed intelligence was changeable had children who were significantly more persistent and achieved higher reading levels than those whose parents believed that intelligence was a fixed, unchangeable trait. This appears to be a relative strength of deaf parents, many of whom reported feeling optimistic about their children’s futures.

Most deaf parents in this sample felt moderately or extremely confident in their parenting overall (82%, see Figure 1). This mirrors previous findings in different samples (Canadian deaf parents: Mallory et al., 1992; disabled vs. nondisabled American parents, including a subset of Deaf parents: Olkin et al., 2006). Despite potential barriers they may face (e.g., see Theme 4), deaf parents in this sample and in previous studies appear to develop strategies that support their overall confidence. This is an important avenue for future research, as both hearing and deaf parents will undoubtedly face challenges in parenthood that could undermine their confidence in themselves.

Overall, many deaf parents reported feeling confident and proud about their children’s educational attainment, optimistic about the future, and confident in their own parenting skills. Notably, support from family, friends, and the wider community was often cited as a reason why many parents felt confident in their parenting and capable of overcoming obstacles (see more in Theme 2 below). It seems feasible to suggest that, as a community who value supporting each other and sharing accessible information, deaf parents might be quick to identify what they need to know or do and to engage their support networks to these ends. Future research might investigate the specific ways that deaf parents leverage community support and shared knowledge to foster resilience and navigate the challenges of parenting. Understanding the mechanisms that bolster deaf parents’ confidence could lead to practical changes that help all parents, whether deaf or hearing, to feel confident in their abilities.

### Theme 2: it takes a village

It appears ubiquitous, in this study and in those previous, that deaf parents rely on those around them for information, practical help, and emotional support. Many specifically mentioned support from the deaf community, which is perhaps unsurprising given significant literature suggesting many deaf people rely on and provide support to others in the deaf community (Holcomb, 2023; Ladd, 2003, 2005; Padden and Humphries, 1988, 2005). As a minority in a hearing world, Deaf people generally value collective community empowerment (Hamill and Stein, 2011) and tend to share accessible information (Holcomb, 2023; Leigh and O’Brien, 2019). Deaf parents in this sample described relying on local deaf communities and online parenting resources in sign language for information about pregnancy, parenting, and child development. As do hearing parents, they likely model successful parenting strategies for each other, seek emotional support from those with similar experiences to their own, or enjoy unimpeded communication to vent their feelings and frustrations to those who will understand.

Research with hearing parents has shown that support from family and friends also plays a role in supporting the psychological transition into parenthood (Angley et al., 2014; Bost et al., 2004) and that being part of a community of other parents is related to feeling in-control, confident, and included in a community (Kane et al., 2007). Again, among hearing samples, parenting groups are thought to reduce significantly parents’ levels of anxiety, stress, anger, and guilt, and increase feelings of empathy and confidence (Barlow et al., 2012, 2016). Levels of social support available to parents has been linked to parents’ beliefs that they can be successful (Fierloos et al., 2023). One study with hearing participants found that mothers who received information about parenthood, as well as both practical and emotional support, were less likely to demonstrate symptoms of postnatal depression at 6 weeks post-delivery (Leahy-Warren et al., 2011). Future research dedicated to this topic with deaf parents could provide clarity about exactly how deaf parents support each other, as well as yield potential strategies from which all parents can learn. More research is needed about the impact of different types of support (e.g., informational, practical, emotional) and its sources (e.g., hearing, deaf, family, friends) for deaf parents. This is particularly important given that previous research has found that the impact of social support for parents is likely related to where it comes from (e.g., from one’s mother versus from one’s friends; Suzuki, 2010).

Importantly, some deaf parents in this sample did not feel they had sufficient social support networks. One deaf parent said that after searching for support groups on social media specific to deaf parents in the UK, and finding none, they decided to create their own. Modern technology affords parents with internet access the opportunity to connect remotely. The availability of face-to-face support varies dramatically depending on where parents live. Several parents who lived where there were large deaf communities (e.g., Austin, Texas, US and Derbyshire, UK) said they could find deaf-specific parenting groups or that they naturally met other deaf parents in day-to-day life. The same was not always true for those living in small, rural towns.

### Theme 3: bimodal bilingualism

Overall, the results suggest that many deaf parents were invested in their children becoming confident bimodal bilinguals. Because some deaf parents had at least one deaf child (*n* = 13) and others had only hearing children (*n* = 24) (see Table 1), descriptions about bilingualism varied. Deaf, signing children are bilingual because they may have some level of access to speech, may use spoken language, and/or because they learn to read a written form of a spoken language (Clark et al., 2020). Hearing children with deaf parents might learn both spoken and sign language. Furthermore, most children of deaf parents in this sample were being raised, regardless of whether the children were hearing or deaf, with some degree of sign language exposure from birth (*n* = 36 of *N* = 37). This means that overall, most children of the parents in this sample were being raised bimodal bilingual. Eight children were exposed to sign language(s) only and *n* = 1 was exposed to spoken English only. It is important to mention that most parents used the general term “bilingual” to refer to their children learning spoken and signed language. There were a variety of specific descriptions (e.g., “sign bilinguals,” “BSL bilinguals”). Here, when we refer to spoken/signed language bilinguals, we use the term “bimodal bilingual” for consistency.

Many were also aware of the importance of providing their children with exposure to high-quality and high-quantity input in both of their languages, and that they devised strategies they felt fit their families best. Strategies tended to focus on providing children with as much exposure as possible to the minority language (sign language). Many strategies were similar to those used by spoken-language bilingual parents. For example, some used the 100-year-old “one parent, one language” approach (De Houwer, 2007; Ronjat, 1913), alternating languages based on location, or changing languages based on the day of the week or time of the day. Some deaf parents also described intentionally introducing their children to other signers which has been shown to be related to larger vocabulary sizes in spoken-language bilingual toddlers (Place and Hoff, 2011).

Some parents were unsure whether their children had sufficient exposure to sign language, given that it is a minority language in a world where spoken language is dominant (De Meulder et al., 2018). Results of the Likert scale ratings also reflected that, for those whose children were learning spoken language, parents were mostly confident about their children’s spoken language development (67% moderately or extremely confident) and less confident overall in their children’s development of sign language (37% moderately or extremely confident). Children’s ages are likely relevant to parents’ feelings: infants who spend all their time with deaf, signing parents will have more daily experience than older children who may be in spoken-language schools and extra-curricular activities for 40 hours each week. Some parents of older children, who spend the majority of their time outside the home described feeling that their children had ample opportunity to learn speech. Research on spoken-language bilinguals has found that children attending school in one language can indeed relate to children becoming less confident in their minority language (De Houwer, 2007). One study by Kanto et al. (2013) found that children of deaf adults’ sign language development was more sensitive to the amount of exposure than was their development of spoken language to spoken language exposure. The authors attributed this result to majority language exposure being more frequent, incidental, and robust than minority sign language exposure. The pattern in the current study implies that many deaf parents might be aware of these trends, and that resources and advice tailored to supporting bimodal bilingualism could be of use.

Crucially, parents’ higher confidence in children’s spoken than sign language development did not mean that deaf parents valued spoken language more than signed language. Many described the importance of easy familial communication overall, whether it be signed and/or spoken. Some referred to specific ways their children mixed and switched between languages while emphasizing that the strategies they have developed worked for their families. It is important for parents to feel confident in their communication with their children, as parent–child language patterns are known to affect the development of children’s language (Rowe, 2012; Hirsh-Pasek et al., 2015), cognition (Pearson et al., 2011; Paavola et al., 2005; Tamis-LeMonda et al., 2001; Smith et al., 2006), and educational outcomes (Cristofaro and Tamis-LeMonda, 2012).

It was common in the present study for deaf parents to feel unsupported in their child’s language development and to encounter people who failed to realize that their child was bilingual. In spoken-language literature, outdated negative attitudes about bilingualism include that bilingual children are “delayed” or “confused” (Guiberson, 2013). Deaf parents in this sample did not describe encountering such overtly negative views about bilingualism, but some deaf parents said that their children were simply not seen as bilingual. The role of sign language in their children’s lives was often overlooked. Several described doctors, midwives, health visitors, and teachers as focussing predominantly on evaluating children’s spoken language milestones. Others said teachers thought their children had language delays because they did not speak as much as same-aged monolinguals, even though sign language was the predominant language at home. Published research has yet to systematically examine the beliefs of mainstream early childhood professionals about bimodal bilingualism (e.g., simultaneous spoken and signed language development). It is possible that those in professions such as pediatric care, health visitation, and early years education, even those with the best intentions, may provide misguided or partially complete advice to deaf parents if they are not sufficiently trained in deaf- and sign-language-related topics. Lack of understanding by professionals could contribute to parents feeling less sure about their children’s bimodal bilingual language development. There is an opportunity both for future research to investigate mainstream attitudes toward bimodal bilingualism and for improvements to continued professional education.

### Theme 4: barriers

Many participants reported that they faced barriers, predominantly of three types: inaccessible information and support, experiences of prejudice and discrimination, and expenditure of extra stress and energy. Challenges were often anchored in contextual factors that parents could not easily control or change, such as the resources of their local governments and the accessibility practices of their local hospitals. What emerged in the data was an overall sense of inequality across deaf parents’ experiences, largely based on uncontrollable and/or random factors, such that some deaf parents did not face challenges in certain domains while others reported many.

Nearly half of the parents in this sample reported some degree of difficulty accessing general parenting support resources, and an even greater percentage – 61% – experienced issues accessing information about pregnancy, parenting, and child development (see Figure 1). Put simply, information is “accessible” when a deaf person is able to expend reasonable effort to engage with it (e.g., Harniss, 2014), though adjustments required to facilitate accessibility depend on the individual. For example, for deaf parents who did not use English as a first language, written English resources about pregnancy, parenting, and child development were often described as inaccessible because they were jargon-heavy. For appointments and events, deaf parents faced barriers to accessible communication without interpreters, which aligns with some existing findings. Research on the medical experiences of deaf childbearing mothers has reported communication difficulties with healthcare providers (in the US: Panko et al., 2023; O’Hearn, 2006; Steinberg et al., 2004; in the UK: Kelsall, 1992; Terry et al., 2024; and in other countries: Gichane et al., 2017). Many deaf parents who requested sign language interpreters were told they could not be booked or that they had been booked, but when they arrived at appointments and events, there were none. This was referenced especially, though not exclusively, for classes run by volunteers or private organizations. Others had interpreters provided who were unqualified and unable to provide full access. Each of these experiences was also reported by Schniedewind et al. (2020) in Deaf signing patients in healthcare settings in Idaho.

For those who wanted them, the impact of lack of sign language interpreters was notable. On an emotional level, several women said they were stressed about securing interpreters for their births. Some said they could find only one interpreter who could not work long lengths of time on their own, while others said they did not have any interpreters for their births at all. Similar results were reported by Terry et al. (2024), who found Welsh deaf mothers experienced long hospital stays without interpreters. One woman in this study said she “had no clue” when her baby was born. There were also practical implications for lack of sign language interpreters for some parents. One attended a baby weighing session without interpreters, and misunderstood information about formula which resulted in an emergency medical appointment. For these parents, lack of sign language interpreters led to gaps in important knowledge that had the potential to affect the health and well-being of both parents and children.

Sign language interpreter provision is considered by many to be a human rights issue (Hauland, 2009), and some countries have equal rights legislation that, if not followed, can have legal implications (e.g., the Equality Act 2010 in the UK and the Americans with Disabilities Act in the US). There is surprisingly little research about the frequency with which deaf people experience barriers to interpreter provision or the effects this might have on health, well-being, or quality of life. It is likely that lack of communication access has both direct and indirect negative effects on deaf people in any situation. For example, one study found that ASL users’ communication with healthcare providers statistically predicted whether their healthcare needs were met (Myers et al., 2021). Another study has reported that written and oral communication, which signers may resort to when interpreters are not provided, was not sufficient to provide Deaf patients with full access to medical information (James et al., 2021). Deaf parents in this study who wanted interpreters reported feeling positively when they were provided, and many said they appreciated when hearing professionals demonstrated sensitivity to deaf people’s communication preferences (as was also reported in Steinberg et al., 2004). Providers who could communicate in sign language (from conversational to fluent proficiency) were appreciated by many deaf parents in this sample, as was also reported in the sample of Steinberg et al. (2004) Deaf American childbearing mothers.

Some deaf parents who did not need sign language interpreters for communication access described how background noise, poor acoustics, and many overlapping speakers made parenting groups inaccessible. Steinberg et al. (2004) reported a similar pattern in prenatal care experiences of deaf women who preferred oral communication to sign language. Despite the fact that oral deaf women did not require a sign language interpreter, they were not more satisfied with their prenatal care than deaf signing women (Steinberg et al., 2004). This could be because, even in situations where sign language interpreters are not required, deaf people may require adjustments to the environment, like rooms with better acoustics, bright lighting, and smaller groups to avoid background noise and overlapping speakers. Failure to address these issues may result in additional barriers to access speech for deaf parents. Organizations and professionals’ relationships with deaf parents could be made easier by increasing deaf awareness and deaf-friendly communication strategies, as well as by providing interpreters for meetings and gatherings.

Many deaf parents also reported continuous encounters with prejudicial attitudes and experiences of discrimination. The root of this problem is that non-deaf people in a hearing-dominant world might view being deaf as a socially devalued, or stigmatized, identity, and those living with stigmatized identities are likely to face unjust social treatment (Mousley and Chaudoir, 2018). For example, some felt medical professionals patronized them, doubted their parenting skills, or questioned whether they should have children at all simply because they were deaf. These patterns align with literature documenting paternalistic attitudes and implicit biases that deaf people often encounter (Ladd, 2003). Several parents also described how professionals appeared ignorant about children’s language development, raising developmentally inappropriate concerns about their child’s spoken language development.

Many deaf parents also said they were treated differently than hearing parents, and some patterns echoed previous reports. Some parents said they received less information than did hearing parents, a finding also reported by O’Hearn (2006) who found Deaf women received significantly less information from doctors than their hearing peers. Deaf parents in this sample also reported inequality in educational settings. One parent said they received noticeably less information about what their child ate and did at nursery than did hearing parents. Deaf parents receiving less information than hearing parents from professionals might be a general phenomenon that occurs both in medical settings as in O’Hearn (2006) and in deaf parents’ interactions with professionals and educators more broadly. Instances of systemic or structural inequality were also prevalent, including booking systems that either required deaf parents to call on the phone (also reported by Terry et al., 2024) or that booked appointments too last-minute for deaf parents to secure interpreters. Taken together, discrimination at both interpersonal and institutional levels reflects broader discussions in the field about the marginalization of deaf people (e.g., Ladd, 2003). There is a need for deaf awareness training to reduce prejudice and interpersonal discrimination, as well as a need to remove institutional barriers in order to make systems more accessible for deaf people.

To overcome common challenges, many deaf parents described working hard and expending extra stress and energy to ensure they were supporting their children optimally. Some deaf parents described the time and energy involved in advocating for their communication needs (e.g., a quieter room, a sign language interpreter), while others described bearing the responsibility of educating professionals about sign language and/or how to engage with deaf people. Similar themes were described in a recent study by VanPuymbrouck and Magasi (2024) that examined disabled people’s experiences about making decisions to request accommodations in healthcare settings. One of the main themes was that of “self-perceived burden,” whereby disabled participants took on extra work because of inaccessible or unaccommodating situations (VanPuymbrouck and Magasi, 2024). Deaf parents described something similar, such as one parent who said that they needed to re-advocate for their needs every year as their children changed classrooms. Another theme reported by VanPuymbrouck and Magasi (2024) was that of “advocacy fatigue,” a term originally coined by Basas (2015) to refer to the “increased strain on emotional, physical, material, social, and wellness resources that comes from continued exposure to system inequities and inequalities,” (p. 39). VanPuymbrouck and Magasi (2024) found that disabled people made careful judgments about the necessity of the accommodation, how likely they were to secure it successfully, and whether they felt prepared to handle experiences of discrimination that could arise as the result of advocating for their needs. Similarly, deaf parents here said they often needed to re-contact and re-request services before potentially securing the accommodations they needed.

### Future directions

There are several limitations of the current study that represent promising opportunities for future research. All participants of this study were residents of Western, educated, industrialized, rich, and democratic countries (‘WEIRD’ countries, Rad et al., 2018). The insights from this work provide a glimpse into the experiences of the deaf parents in the sample but do not represent the experiences of all deaf parents, even those from WEIRD countries. Even less so do the results represent the experiences of deaf parents from the non-Western, less-industrialized, poorer, or nondemocratic countries (Muthukrishna et al., 2020). Future research should continue to work with non-WEIRD samples of deaf parents (e.g., Almohsin et al., 2023; Çoksan et al., 2024; Gichane et al., 2017; Suzuki, 2010) so that the literature represents the full diversity of deaf parents’ experiences around the world.

Most deaf parents in this study used some degree of sign language with their children. Only one parent used only speech. Future research may fill this gap by investigating specifically the lived experiences of deaf parents who use only spoken language. Mothers were also more strongly represented than fathers, and those from two-parent households were more prevalent than were single parents (see Table 1). Overall, the composition of this sample is important both to the interpretation of the results of the thematic analysis broadly (Braun and Clarke, 2022) and to research with deaf people specifically given their broad diversity of audiological, social, cultural, and linguistic experiences (Allen, 2015). For example, it was obvious in participants’ responses that many experiences were location-dependent; parents often referenced access to resources in their specific towns/villages – whether they were rural versus urban, whether they had access to high-quality/varied healthcare, and whether they were nearby other deaf people. However, it is also likely that the broader social-political landscape in participants’ countries plays a part in parents’ access to resources. Future cross-cultural research might investigate what elements of a deaf parents’ immediate environment can facilitate positive experiences and whether sources of support are similar or different across countries. Overall, future research may endeavor to explore the lived experiences of deaf parents with different identities, language preferences, and family situations that are not well-represented here.

The inductive thematic analysis methodology applied here took a bottom-up approach to construct themes rather than to impose or test a strict theoretical framework (Braun and Clarke, 2022). However, a full picture of deaf parents’ experiences would be best served by using a broad array of other qualitative methods, such as inductive/deductive hybrid methods (Proudfoot, 2022) or by using narrative analysis to understand how individuals understand their own experiences (McLeod, 2024).

In addition to different methodologies, it is critical that future research be conducted by scientists with diverse identities and positions. Reflexive thematic analysis uses researchers’ subjectivity as a tool in the transparent construction of meaning from a dataset (Braun and Clarke, 2022). By definition, a single reflexive thematic analysis can only ever offer the field the insight of that one team’s experiences. The true diversity of deaf parents’ experiences can only be fully understood if future research is led by teams who bring a range of intersecting identities (e.g., race, gender, age, class, culture, geographic location) and methodological approaches. Encouraging diversity in research teams contributes directly to the generation of new insights using qualitative analysis.

For example, this research teams’ involvement in social-cultural deaf networks shaped recruitment and survey question construction. Survey questions were available in either English or sign language (ASL or BSL), and there was an option to take part in a live focus group with sign language interpreters provided. The recruitment structure likely incentivized deaf signers to take part, perhaps contributing to the majority of this sample having children who were exposed to some degree of sign language (see Table 1). It is also notable that this research was inspired by the team’s experiences working with deaf parents of young children who often described issues of accessibility in medical settings and, later in life, how bimodal bilingualism was misunderstood by professionals. The survey questions asked parents to describe their experiences in these areas, which likely led to their description of specific anecdotes. Perhaps because of the nature of the questions (see Appendix), there was not a strong focus on the broad ways in which deaf parents’ cultural identities shaped their experiences in parenting/early childhood contexts. For example, it might be that deaf parents from deaf families rely on support from deaf family/community and therefore do not experience barriers to the same degree as deaf parents from hearing families. Future research may fill this gap by asking deaf parents in detail about their language experiences, culturally identities, and whether (or how) these factors influence their engagement with early childhood contexts. Such research also stands to identify factors that contribute to resilience in the face of discriminatory experiences.

It is also important to reiterate that all three members of the research team were either outsiders or insider-outsiders in the sample studied (Dwyer and Buckle, 2009). Their various identities will have influenced their development of the research questions, data collection and analysis, and writing of this manuscript. It has been suggested that, in qualitative investigation, outsiders may approach research questions in ineffective ways or may omit questions that an insider would have thought to ask (Kerstetter, 2012). Group insiders might hold certain beliefs about research conducted by outsiders and insider-outsiders, which could have affected the information they shared and/or whether they chose to participate in the study at all (Dwyer and Buckle, 2009). There may also be benefits of the fresh perspectives that outsiders and insider-outsider researchers bring to the research process. Looking ahead, it is critical that there be future research about the lived experiences of deaf people that is led by deaf people. The importance of deaf-led research has been discussed at length in other fields, such as that of Deaf studies (Kusters et al., 2017; O’Brien and Emery, 2013). Working toward a truly inclusive field of qualitative research on the lived experiences of deaf parents will best serve the academic literature, as well as the communities it seeks to understand.

## Conclusion

Despite facing some barriers, deaf parents in this study described themselves as hard-working, confident in themselves, and proud of their children. They were impressed by their children’s school achievements and optimistic about their prospects for future success. Many also felt confident in their parenting skills, which were often described as related to the informational and practical support of their friends, family, and wider communities. Some parents felt there were no strong, obvious networks of deaf parents for them to connect with, which could lead to feelings of isolation and exclusion. Most parents were raising their children as bimodal bilinguals, enacting specific language strategies to bolster their sign language development in particular. Finally, deaf parents faced several common challenges. There is a clear need to prioritize accessible information, resources, and support groups for deaf parents, though doing so can benefit all parents. Particularly important for deaf parents is consistent interpreter provision and/or making accommodations to the environment, depending on the needs of the individual. Many deaf parents advocated for more specialized resources and deaf- and bimodal bilingual-specific training for midwives, doctors, and teachers. Increasing deaf awareness among those who work with parents could reduce instances of prejudice and discrimination to foster more equitable and inclusive experiences of parenthood.

## Data Availability

The raw data supporting the conclusions of this article will be made available by the authors, without undue reservation.

## Appendix

### List of Survey Questions

1. How easy has it been for you to find accessible information about pregnancy, parenting, and child development?
  - Please use this space to tell us more.
2. How easy has it been to find accessible parenting support?
  - Please use this space to tell us more.
3. How much support have you felt from healthcare staff?
  - Please use this space to tell us more.
4. How much support have you felt from nursery/school or other educational staff?
  - Please use this space to tell us more.
5. Did you receive any input from medical staff about your children’s language development?
  - If yes, how helpful was this input?
6. Did you receive any input from nursery/school or other educational staff about your child’s language development?
  - If yes, how helpful was this input?
7. How confident are you feeling as a parent?
  - Please use this space to tell us more.
8. How confident are you about your children’s sign language development (if relevant)?
  - Please use this space to tell us more.
9. How confident are you about your children’s spoken language development (if relevant)?
  - Please use this space to tell us more.
10. How confident are you about your children’s school integration and achievements?
  - Please use this space to tell us more.
11. In what areas would you like to see more information about the development of children with deaf parents?
